# Efficient Probabilistic and Geometric Anatomical Mapping Using Particle Mesh Approximation on GPUs

**DOI:** 10.1155/2011/572187

**Published:** 2011-08-17

**Authors:** Linh Ha, Marcel Prastawa, Guido Gerig, John H. Gilmore, Cláudio T. Silva, Sarang Joshi

**Affiliations:** ^1^Scientific Computing and Imaging Institute, University of Utah, Salt Lake City, UT 84112, USA; ^2^Department of Psychiatry, University of North Carolina, Chapel Hill, NC 27599, USA

## Abstract

Deformable image registration in the presence of considerable contrast differences and large size and shape changes presents significant research challenges. First, it requires a robust registration framework that does not depend on intensity measurements and can handle large nonlinear shape variations. Second, it involves the expensive computation of nonlinear deformations with high degrees of freedom. Often it takes a significant amount of computation time and thus becomes infeasible for practical purposes. In this paper, we present a solution based on two key ideas: a new registration method that generates a mapping between anatomies represented as a multicompartment model of class posterior images and geometries and an implementation of the algorithm using particle mesh approximation on Graphical Processing Units (GPUs) to fulfill the computational requirements. We show results on the registrations of neonatal to 2-year old infant MRIs. Quantitative validation demonstrates that our proposed method generates registrations that better maintain the consistency of anatomical structures over time and provides transformations that better preserve structures undergoing large deformations than transformations obtained by standard intensity-only registration. We also achieve the speedup of three orders of magnitudes compared to a CPU reference implementation, making it possible to use the technique in time-critical applications.

## 1. Introduction

**Figure 1:**
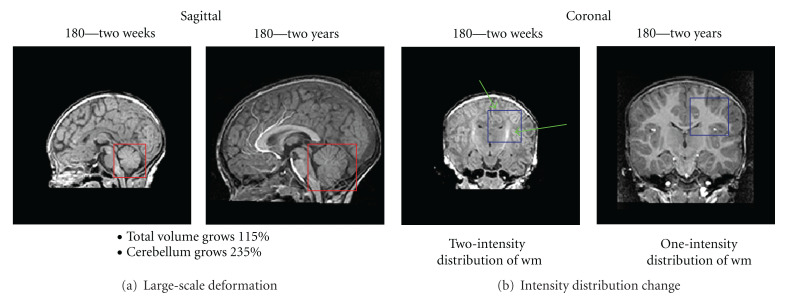
Registration challenges of human brains at early development stages. The image show significant shape and size changes of an infant brain of subject 180 from two weeks to two years as well as the changing white matter properties and appearance due to the myelination.

Our work is motivated by the longitudinal study of early brain development in neuroimaging, which is essential to predict the neurological disorders in early stages. The study, however, is challenging due to two primary reasons: the large-scale nonlinear shape changes (the image processing challenge) and the huge amount of computational power the problem requires (the computational challenge). The image processing challenge involves robust image registration to define anatomical mappings. While robust image registrations have been studied extensively in the literature [1–3], registration of the brain at early development stage is still challenging as the growth process can involve very large-scale size and shape changes, as well as changes in tissue properties and appearance (Figure 1). Knickmeyer et al. [4] showed that the brain volume grows by 100% the first year and 15% the second year, whereas the cerebellum shows 220% volume growth for the first and another 15% for the second year. These numbers indicate very different growth rates of different anatomical structures. Through regression on shape representations, Datar et al. [5] illustrated that the rapid volume changes are also paralleled by significant shape changes, which describe the dynamic pattern of localized, nonlinear growth. A major clinical research question is to find a link between cognitive development and the rapid, locally varying growth of specific anatomical structures. This requires registration methods to handle large-scale and also nonlinear changes. Also, the process of white matter myelination, which manifests as two distinct white matter appearance patterns primarily during the first year of development, imposes another significant challenge as image intensities need to be interpreted differently at different stages.

To approach these problems, a robust registration method is necessary for mapping longitudinal brain MRI to a reference space so that we can perform reliable analysis of the tissue property changes reflected in MR measurements. This method should not rely on raw intensity measurements, while it should be capable of estimating large structural deformations. Xue et al. [6] addressed these issues by proposing a registration scheme for neonatal brains by registering inflated cortical surfaces extracted from the MRI. Their registration method does not make use of voxel-wise image information and is not intended to capture growth in internal structures. It is designed for analyzing cortical surfaces, and it does not define a transformation for the whole brain volume.

In this paper, we propose a new registration framework for longitudinal brain MRI that makes use of underlying anatomies, which are represented by geometries and class posterior images. This framework can match internal regions and simultaneously preserve a consistent mapping for the boundaries of relevant anatomical objects. We show results of registering neonatal brain MRI to 2-year old brain MRI of the same subjects obtained in a longitudinal neuroimaging study. Our method consistently provides transformations that better preserve time-varying structures than those obtained by intensity-only registration [7].

The study presents a significant computational challenge because dense, free-form mapping is computationally expensive. In particular, a correspondence-free geometric norm such as “currents” has computational complexity of *O*(*M*^2^) where *M* is the number of geometric elements, which is in the same order of the image volume [8]. These methods require supercomputing power to run [9], but still take a considerable amount of time to complete. While access to a supercomputer system or even a cluster is not available to most researchers, robust registration in the presence of large deformations is essential. Fortunately, this computation problem finds an economical solution via the work of High-Performance Computing (HPC) General Processing on Graphical Processing Units (GPUs) community. Modern GPUs, which are available on commodity hardware, could offer several teraflops of peak performance, which is equivalent to that of a super computer in the mid-90s. There have been a number of image processing applications being implemented on GPUs [10–13]. Most applications achieve from 20x to several magnitudes of speedup when moved to GPUs in comparison to conventional CPU versions. A closely related example is the fast Greedy Iterative Diffeomorphic registration framework by Ha et al. [14] using GPUs that achieved 60x speedup in comparison to an optimized, fully parallel version running on an eight-core Xeon 3.2 Ghz sever.

However, mapping algorithms from the CPU to the GPU is nontrivial. The GPU programming model is significantly different from the CPU programming model. While GPUs are highly efficient for parallel data processing, they are slow for serial scalar code, which exists in any processing algorithms. To achieve a high performance, it often requires developers to reformulate the problem so that it is mapped well to the GPU architecture. In this paper, we present the implementation of our registration framework on commodity GPUs. We introduce two primary performance improvements with a combination of two approaches: (1) an algorithmic improvement using a particle mesh approach and (2) parallelisation using GPUs. We are able to solve the practical problem in real time and gain speedup of nearly three magnitudes order over CPU reference implementation. 

## 2. Related Work

The development of image registration is the major focus of computational anatomy [3, 15–17]. There are two large bodies of research that our method is developed on: large deformation diffeomorphic registration and multicompartment registration via surface matching.

The analysis of shape and size in anatomical images models anatomy as a deformable template [18]. Common image registration techniques based on thin-plate splines and linear-elastic models [19, 20] have a small deformation assumption and cannot be used due to the large localized deformations associated with early brain development. The large deformation model for computing transformations developed by Christensen et al. [21] overcomes the limitations of the small deformations model by ensuring that the transformations computed between imagery are diffeomorphic (smooth and invertible). Based on the large deformation framework by Miller and Younes [3], Beg et al. [22] derived the Large Deformation Diffeomorphic Metric Mapping (LDDMM) algorithm. This method computes an optimal velocity field that satisfies the Euler-Lagrange variational minimization constraints. Our method is developed upon the greedy approach proposed by Christensen et al. [21] that often reports high registration quality comparable to LDDMM approach but requires significantly lower amount of computation. 

Surface matching is usually considered a semiautomatic procedure and a “point correspondence” task. First, a small number of anatomical features such as landmark points and curve are identified by hand. Next, each of these features of the discretized surface finds its corresponding feature on the target. This matching information is then used to guide the transformation of the entire surface [19, 23, 24]. This approach, however, has a fundamental issue due to discretization. The currents distance was introduced by Vaillant and Glaunès [25] as a way of comparing shapes (point sets, curves, surfaces) without having to rely on computing correspondences between features in each shape.

Most of the current registration techniques currently being used in computational anatomy are based on single-subject anatomy [18, 25–27]. This approach is limited since a single anatomy cannot faithfully represent the complex structural variability and development of the subjects. Our method is based on the multicompartment model proposed by Glaunes and Joshi [28] which defines a combined measurement acting on different anatomical features such as point, curve, and surface to enhance registration quality.

Existing works refer to computational anatomy, especially free-from matching, as a robust but computationally expensive framework which is difficult to achieve in real time on commodity hardware [9, 29, 30]. In this paper, we consider GPU implementation as an integral part of our work and an essential contribution that allows scientists to accurately register images and geometries in time-critical applications. 

## 3. Method

**Figure 2:**
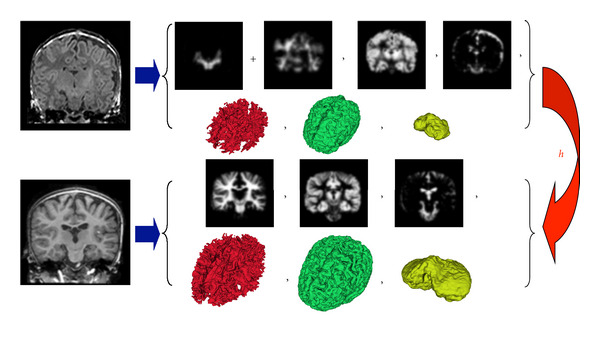
Overview of the proposed registration method that can handle large deformations and different contrast properties, applied to mapping brain MRI of neonates to 2-year olds. We segment the brain MRIs and then extract equivalent anatomical descriptors by merging the two different white matter types present in neonates. The probabilistic and geometric anatomical descriptors are then used to compute the transformation *h* that minimizes the distance between the class posterior images, as well as the distance between surfaces represented as currents.

We propose a new registration method that makes use of the underlying anatomy in the MR images.  Figure 2 shows an overview of the registration process. We begin by extracting probabilistic and geometric anatomical descriptors from the images, followed by computing a transformation that minimizes the distance between the anatomical descriptors. 

### 3.1. Anatomical Descriptors

We represent brain anatomy as a multicompartment model of tissue class posteriors and manifolds. We associate each position *x* with a vector of tissue probability densities. In a given anatomy, we capture the underlying structures by estimating, for each image, the class posterior mass functions associated with each of the classes. Given Ω as the underlying coordinate system of the brain anatomies, each anatomy *𝒜*_*i*=1,…,*N*_ is represented as

(1) $$\begin{array}{c} {𝒜}_{i}=\left\{p_{i,c=1}\left(x\right),\ldots ,p_{i,c=N_{c}}\left(x\right),{ℳ}_{i,j=1}\left(2\right),\ldots ,{ℳ}_{i,j=N_{s}}\left(2\right)\subset \Omega \right\}, \end{array}$$

where *N*_*c*_ is the number of probability images, *N*_*s*_ is the number of surfaces, *p*_*c*_(*x*) is the class posterior for tissue *c* at location *x*, and *ℳ*_*j*_(2) are 2-dimensional submanifolds of Ω (surfaces).

As we are interested in capturing major growth of the white matter and gray matter growth, we represent brain anatomy as a tuple of the probabilities {*p*_wm_(*x*), *p*_gm_(*x*), *p*_csf_(*x*)} representing class posterior probabilities of white matter, gray matter, and cerebrospinal fluid respectively, followed by the surfaces of white matter, gray matter, and cerebellum.

The classification of brain MR images with mature white matter structures into class posteriors is well studied. We extract the posteriors from 2-year old brain MR images using the segmentation method proposed by van Leemput et al. [31]. The method generates posterior probabilities for white matter (wm), gray matter (gm), and cerebrospinal fluid (csf). These probabilities can then be used to generate surfaces from the maximum a posteriori tissue label maps.

The classification of neonatal brain MR images is challenging as the white matter structure undergoes myelination, where the fibers are being covered in myelin sheathes. Several researchers have proposed methods that make use of prior information from an atlas or template that takes into account the special white matter appearance due to myelination [32]. We use the method described by Prastawa et al. [33] for extracting the tissue class posteriors of neonatal brain MRI, which includes for myelinated wm, nonmyelinated wm, gm, and csf. These can then be used to create an equivalent anatomy to the 2-year old brain by combining the two white matter class probabilities which then leads to a single white matter surface.

The white matter and gray matter surfaces are generated from the maximum a posteriori (MAP) segmentation label maps using the marching cubes algorithm [34]. The cerebellum surfaces are generated from semiautomated segmentations that are obtained by affinely registering a template image followed by a supervised level set segmentation. The cerebellum has a significant role in motor function, and it is explicitly modeled as it undergoes the most rapid volume change during the first year of development and thus presents a localized large-scale deformation. 

### 3.2. Registration Formulation

Given two anatomies *𝒜*_1_ and *𝒜*_2_, the registration problem can be formulated as an estimation problem for the transformation *h* that minimizes

(2) $$\begin{array}{c} \hat{h}=\underset{h}{\text{argmin}}\;\;E{\left(h\cdot {𝒜}_{1},{𝒜}_{2}\right)}^{2}+D{\left(h,e\right)}^{2}\;, \end{array}$$

where *h* · *𝒜*_1_ is the transformed anatomy, *E*(·, ·) is a metric between anatomies, and *D*(·, *e*) is a metric on a group of transformations that penalizes deviations from the identity transformation *e*. The anatomy is transformed using backward mapping for probability image and forward mapping for geometries:

(3) $$\begin{array}{cc} h\cdot {𝒜}_{1} & =h\cdot \{p_{i,c=1}\left(x\right),\ldots ,p_{i,c=N_{c}}\left(x\right),\\ & \;\;\;{ℳ}_{i,j=1}\left(2\right),\ldots ,{ℳ}_{i,j=N_{s}}\left(2\right)\}\\ & =\{p_{i,c=1}\left(x\right)\circ h^{-1},\ldots ,p_{i,c=N_{c}}\left(x\right)\circ h^{-1},\\ & \;\;h\left({ℳ}_{i,j=1}\left(2\right)\right),\ldots ,h\left({ℳ}_{i,j=N_{s}}\left(2\right)\right)\}. \end{array}$$

We define distance between anatomies *E* by defining a norm on an anatomy as a combination of the *L*^2^ norm on the class posteriors and a Reproducing Kernel Hilbert space norm on the manifolds defined as “currents” through Glaunes et al. [1]. This norm does not require prior knowledge on geometric correspondence, as compared to other geometry matching methods [19, 23, 24] that require explicit specification of geometric correspondences. More precisely, they require that a certain point *q* in object A is the same (anatomically) as point *q* in object B; hence, object A and object B are required to have the same number of elements and the same ordering of elements. In comparison, the currents norm defines distance between objects based on the norm measurement of the union of the geometric objects. The currents norm is thus correspondence-free and does not require the objects in comparison to have equal number of elements and the same ordering or anatomical definition.

In contrast to Iterative Closest Point (ICP) algorithm [35] which defines correspondence based on the closest features on the Euclidean space, the currents matching algorithm compares each element to all other elements. Since there may not exist an anatomically homologous correspondence for every feature due to discretization, the currents matching is more robust than existing methods. Manifolds with different number of elements (resolutions) can thus be matched using the currents norm due to this property. For an oriented surface *ℳ*(2) in *R*^3^ the norm [*ℳ*(2)] is the vector-valued Borel measure corresponding to the collection of unit normal vectors to *ℳ*(2), distributed with density equal to the element of surface area *ds* and can be written as *η*(*x*)*ds*(*x*), where *η*(*x*) is the unit normal and *ds*(*x*) is the surface measure at point *x*. The currents representation forms a vector space that admits linear operations, unlike other surface representations such as the Signed Distance Map [36–38].

Given an anatomy *𝒜* the *k*-norm of [*𝒜*] is composed as

(4) $$\begin{array}{c} {||\left[𝒜\right]||}_{k}^{2}={||P\left(x\right)||}_{L^{2}}+{||\left[ℳ\left(2\right)\right]||}_{k}, \end{array}$$

where the probabilistic norm is defined as

(5) $$\begin{array}{cc} {||P\left(x\right)||}_{L^{2}} & =\overset{N_{c}}{\underset{c=1}{\sum }}{||p_{1,c}\left(x\right)-p_{2,c}\left(x\right)||}_{k}^{L^{2}}\\ & =\int_{\Omega }{\left|p_{1,c}\left(x\right)-p_{2,c}\left(x\right)\right|}^{2}dx \end{array}$$

and the currents norm is given by

(6) $$\begin{array}{c} \begin{array}{c} {||\left[ℳ\left(2\right)\right]||}_{k} \end{array} \end{array}=\iint_{ℳ\left(2\right)}^{}k\left(x,y\right)\langle \eta \left(x\right),\eta \left(y\right)\rangle d\mu \left(x\right)d\mu \left(y\right),$$

where *k*(·, ·) is a shift-invariant kernel (e.g., Gaussian or Cauchy).

When *ℳ*(2) is a discrete triangular mesh with *N*_*f*_ faces, a good approximation of the norm can be computed by replacing [*ℳ*(2)] by a sum of vector-valued Dirac masses

(7) $$\begin{array}{c} {||\left[ℳ\left(2\right)\right]||}_{k}^{2}=\overset{N_{f}}{\underset{f=1}{\sum }}\overset{N_{f}}{\underset{f\prime =1}{\sum }}{\langle \eta \left(f\right),\eta \left(f^{\prime }\right)\rangle }_{}k\left(c\left(f\right),c\left(f^{\prime }\right)\right), \end{array}$$

where *N*_*f*_ is the number of faces of the triangulation and, for any face *f*, *c*(*f*) is its center and *η*(*f*) its normal vector with the length capturing the area of each triangle.

Having defined the norm on probability images and surfaces, the dissimilarity metric between anatomies ||[*𝒜*_1_]−[*𝒜*_2_]||_*k*_^2^ is given by

(8) $$\begin{array}{cc} & w_{p}\overset{N_{c}}{\underset{c=1}{\sum }}{||p_{1,c}\left(x\right)-p_{2,c}\left(x\right)||}_{k}^{L^{2}}+w_{g}\overset{N_{s}}{\underset{j=1}{\sum }}{||\left[{ℳ}_{1,j}\left(2\right)-{ℳ}_{2,j}\left(2\right)\right]||}_{k}^{2}\\ & \;\;=w_{p}\overset{N_{c}}{\underset{c=1}{\sum }}\int_{\Omega }{\left|p_{1,c}\left(x\right)-p_{2,c}\left(x\right)\right|}^{2}dx\\ & \;\;\;+w_{g}\overset{N_{s}}{\underset{j=1}{\sum }}{||\left[{ℳ}_{1,j}\left(2\right)\cup \left(-{ℳ}_{2,j}\left(2\right)\right)\right]||}_{k}^{2}, \end{array}$$

where the distance between two surface currents ||[*ℳ*_1,*j*_(2)−*ℳ*_2,*j*_(2)]||_*k*_ = ||[*ℳ*_1_(2)∪(−*ℳ*_2_(2))]||_*k*_ is computed as the norm of the union between surface *ℳ*_1_(2) and surface *ℳ*_2_(2) with negative measures, *w*_*p*_ and *w*_*g*_ are scalar weights that balance the influence of probabilistic and geometric presentations.

We use the large deformation framework [3] that generates dense deformation maps in *R*^*d*^ by integrating time-dependent velocity fields. The flow equation is given by ∂*h*^*v*^(*t*, *x*)/∂*t* = *v*(*t*, *h*^*v*^(*t*, *x*)), with *h*(0, *x*) = *x*, and we define *h*(*x*)∶ = *h*^*v*^(1, *x*), which is a one-to-one map in ℝ^*d*^, that is, a diffeomorphism. The diffeomorphism is constructed as a fluid flow that is smooth and invertible. The invertibility of the mapping is a desirable property as it enables analysis in different spaces and time points as needed. We define an energy functional that ensures the regularity of the transformations on the velocity fields: ||*v*(*t*,·)||_*V*_^2^ = ∫_ℝ^*d*^_〈*Lv*(*t*,*x*),*Lv*(*t*,*x*)〉*dx*, where *L* is a differential operator acting on vector fields. This energy also defines a distance in the group of diffeomorphisms:

(9) $$\begin{array}{c} D^{2}\left(h,e\right)=\underset{v,p^{v}\left(1,\cdot \right)=h}{\inf\;}\overset{1}{\underset{0}{\int }}{||Lv\left(t\right)||}_{V}^{2}dt. \end{array}$$

The registration optimizations in this paper are performed using a greedy approach by iteratively performing gradient descent on velocity fields and updating the transformations via an Euler integration of the O.D.E. At each iteration of the algorithm the velocity field is calculated by solving the PDE:

(10) $$\begin{array}{c} Lv=F\left(h\right), \end{array}$$

where *v* is the transformation velocity field, *L* = *α*∇^2^ + *β*∇·∇+*γ*, and *F*(*h*) is the variation of ||[*h* · *𝒜*_1_]−[*𝒜*_2_]||_*k*_^2^ with respect to *h*. This variation is a combination of the variation of the *L*^2^ norm on the class posteriors and of the currents norm, computed using the gradient

(11) $$\begin{array}{cc} \frac{\partial {||\left[ℳ\left(2\right)\right]||}_{k}^{2}}{\partial x_{r}} & =\underset{f|x_{r}\in f}{\sum }\left[\frac{\partial \eta \left(f\right)}{\partial x_{r}}\right]\overset{N_{f}}{\underset{f\prime =1}{\sum }}k\left(c\left(f^{\prime }\right),c\left(f\right)\right)\eta \left(f^{\prime }\right)\\ & \;+\frac{2}{3}\overset{N_{f}}{\underset{f\prime =1}{\sum }}\frac{\partial k\left(c\left(f\right),c\left(f^{\prime }\right)\right)}{\partial c\left(f\right)}\eta {\left(f^{\prime }\right)}^{t}\eta \left(f\right), \end{array}$$

given that points {*x*_*r*_, *x*_*s*_, *x*_*t*_} form the triangular face *f* and its center *c*(*f*) = (*x*_*r*_ + *x*_*s*_ + *x*_*t*_)/3 and its area-weighted normal *η*(*f*) = (1/2)(*x*_*s*_ − *x*_*r*_)⊗(*x*_*t*_ − *x*_*r*_).

The currents representation is generalized to account for not only surface meshes but also other *m*-submanifolds such as point sets or curves. The currents associated to an oriented *m*-submanifold *ℳ* is the linear functional [*ℳ*] defined by [*ℳ*](*ω*) = ∫_*ℳ*_*ω*. When *ℳ*(0) = ⋃*x*_*i*_ is a collection of points [*ℳ*(0)] is a set of Dirac delta measures centered at the points that is, [*ℳ*(0)] = ∑_*i*_*α*_*i*_*δ*(*x* − *x*_*i*_). When *ℳ*(1) is a curve in ℝ^3^, [*ℳ*(1)] is the vector-valued Borel measure corresponding to the collection of unit-tangent vectors to the curve, distributed with density equal to the element of length *dl*:

(12) $$\begin{array}{c} {||\left[ℳ\left(1\right)\right]||}_{k}^{2}=\overset{N_{l}}{\underset{l=1}{\sum }}\overset{N_{l}}{\underset{l^{\prime }=1}{\sum }}{\langle \tau \left(l\right),\tau \left(l^{\prime }\right)\rangle }_{}k\left(c\left(l\right),c\left(l^{\prime }\right)\right), \end{array}$$

where *N*_*l*_ is the number of line segments and, for any segment *l* with vertices *v*_0_ and *v*_1_, *c*(*l*) = (*v*_*o*_ + *v*_1_)/2 is its center and *τ*(*l*) = *v*_1_ − *v*_0_ is its tangent vertor with its length capturing the length of the line segment.

Using extra submanifold presentation helps capture important properties of the target anatomy and hence could potentially direct the registration and improve the result; see Glaunes et al. [1] for more details. 

## 4. Efficient Implementation

The implementation of our registration framework is based on two critical sections: large deformation diffeomorphic image registration and currents norm computation. The former requires a linear solver (10) on an *M* × *M* matrix where *M* is the number of input volume elements (*≈*10 millions on typical brain image). The linear system is sparse and there exists efficient solver with complexity of *O*(*M*log (*M*)). The performance is even further amortized using a multiscale iterative method resembling a multigrid solver. The method maps well to the GPU architecture and significantly reduces the running time from several hours on eight-core sever to a few minutes on commodity hardware. We refer to the work by Ha et al. [14] for details of the method and implementation of large deformation diffeomorphic registration on GPUs. Here, we concentrate on the problem of how to implement norm computation efficiently based on GPU methodologies.

At a broad level, the GPUs consist of several streaming multiprocessors—each of them contains a number of streaming processors and a small shared memory unit. GPUs are good at handling data stream in parallel with processing kernels [39]. The underlying program structure is described by streams of data passing through computation kernels. Given a set of data (an input stream), a series of operations (kernel functions) are applied to each element in the stream and produce another set of output data (an output stream). The program is constructed by chaining these computations together. This formulation has been used to design efficient GPU-based sorting and numerical computations [14, 40, 41]. 

### 4.1. Particle Mesh Approximation for Currents Norm Computation

The major challenge of computing the currents norm (7) for real brain surfaces is the high computational cost to compute the dissimilarity metric of all pairs of surface elements, which is *O*(*N*_*f*_^2^), where *N*_*f*_ is the number of faces. A surface extracted from an *N*^3^ volume has the average complexity of *N*^2.46^ faces [8], that produces millions surfaces for a typical 256^3^ input.

For computational tractability, Durrleman et al. [42] used a sparse representation of the surface based on matching pursuit algorithm. On the other hand, an efficient framework based on the standard fast Gauss transform [43] requires the construction and maintenance of the kd-tree structures on the fly. The primary problem of these approaches is that while the performance is insufficient for real-time applications on conventional systems, they are too sophisticated to make use of processing power of modern parallel computing models on GPUs. Also in practice, we use large kernel width for the currents norm to match major structures. This is not ideal for kd-tree-based implementations that are designed for querying small set of nearest neighbor. Implementing these ideas on GPUs imposes other challenges, and they are unlikely to be efficient.

Here, we employ a more parallelizable approach based on the Particle Mesh approximation (PM). This approximation has been extensively studied in a closely related problem—the cosmological *N*-body simulation, which requires the computation of the interaction between every single pair of objects (see Hockney and Eastwood [44] for details).

**Figure 3:**
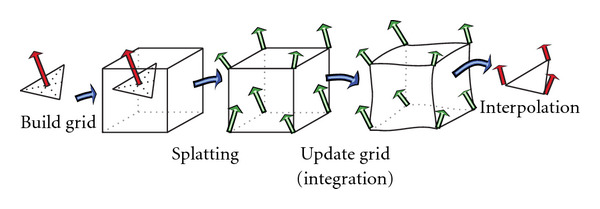
Particle mesh approximation algorithm to transform the computation from irregular domain to regular domain based on four basic steps: grid construction, splatting, integration, and interpolation.

The particle mesh approximation, as shown in Figure 3, includes four main steps.

*Grid building* which determines the discretization error or the accuracy of the approximation. It also specifies the computational grid, the spacial constraints of the computation. The quantization step in each spacial direction determines the grid size, hence, the complexity of the grid computation. The finer the grid means the higher quality of the approximation but the more computation involving.

*Splatting* that maps computation from an unstructured grid to a structured grid. It is the inverse operation of the interpolation.

*Integration* which performs the grid computation and updating step. As the computation, which involves kernel convolution and gradient computation, is taking place in a regular domain, the integration can exploit the parallel processing power of special computing units such as GPUs.

*Interpolation* that interprets computational results from the image space back to the geometrical space, in other words, to reconstruct the unstructured grid out of the structured domain. Marching Cube [34] is an example of techniques using interpolation to extract isosurfaces from MR images.

The splatting/interpolation operation pair works as a connection between the computation on regular domain and irregular domain. We will go into details of how to implement this interface on the parallel architecture as the method can be widely used not only for the norm computation but any mixed—geometric and probabilistic—computation in general. We consider this strategy as a crucial method for efficient parallel computation on an irregular domain.

**Figure 4:**
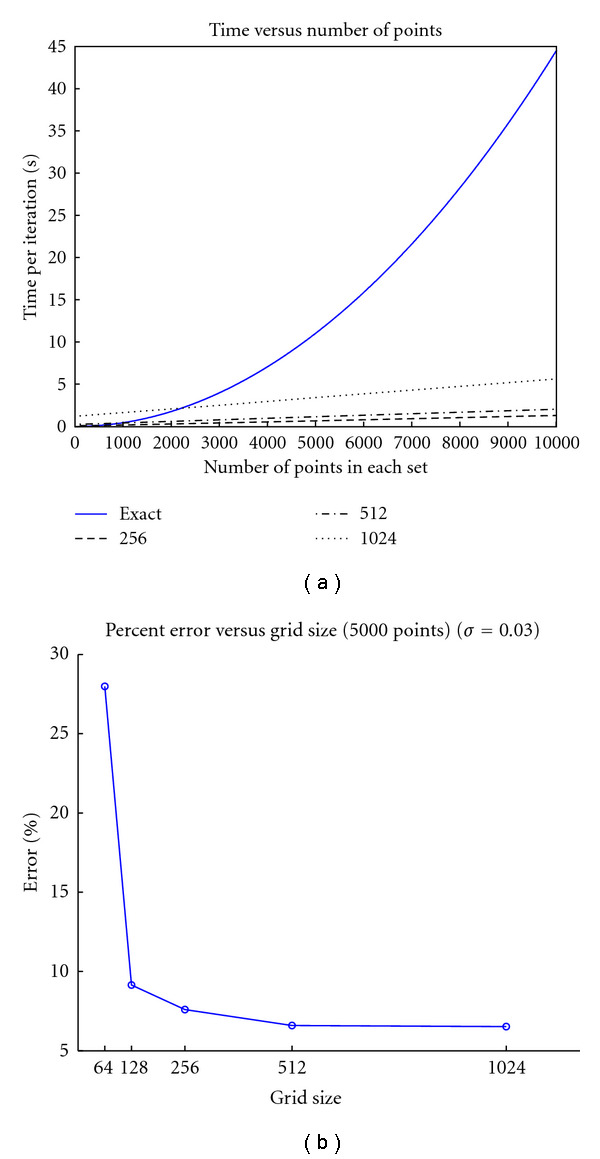
(a) shows the run time comparisons between direct computation and the particle mesh implementation for various grid size. Shown in (b) is the percent error for different for 5000 randomly generated points with different mesh sizes.

The error in particle mesh approximation is influenced by two factors: the grid spacing and the width of the convolution kernel, as shown in Figure 4. We chose the image grid spacing, thus the error is bounded by the image resolution. As being aforementioned, we use large kernel widths in practice which is ideal for PM. Note that PM approximation breaks down when kernel width is less than grid spacing.

While the approximation helps reduce the complexity to *M*log *M* where *M* is the volume size of the embedded grid, the total complexity of the method is still very high. On a high-end workstation with 8-CPU cores, a highly optimized multithreaded implementation in C++ takes several hours for one matching pair hence cannot be used for parameter exploration and real-time analysis. Based on the GPU framework by Ha et al. [14], we developed an implementation that runs entirely on the GPU to exploit parallel efficiency of regular grid presentation. 

### 4.2. Efficient Implementation of Particle Mesh Method on GPUs

To achieve the maximum performance efficiency, we optimized the four steps of particle mesh method on GPUs. Here, we describe the performance keys and important details to implement these steps. 

#### 4.2.1. Grid Building

Without prior information, computational grid is typically chosen as a discretization of the bounding box with extra border regions to prevent out-of-bound quantization error. Since probabilistic and geometric descriptors coexist in our representation, the computational grid is effectively chosen as the original grid. This selection guarantees that it will not introduce further quantization errors than the original discretized errors inherent to the construction of geometric descriptors. This strategy also limits the complexity of the combining technique to the original order of computation if we use only probabilistic terms. 

#### 4.2.2. Splatting

The main purpose of the splatting function is to construct a regular *n*-dimensional scalar or vector field from its discrete sample points. The constructed grid should satisfy an inverse operation, the interpolation, so that when applied to the reconstructed grid will reproduce the sample points. In other words, Interpolation (Splatting (*E*)) = *E* with *E* is an arbitrary input. This duality of splatting and interpolation reflects the fact that probabilistic and geometry descriptors are just the domain representations of the same subject. Hence, we could unify their computation without losing accuracy. We also exploit the duality to validate the correctness of our implementation of the splatting function through its dual counterpart.

The splatting function is defined by Trouvé and Younes [45] through a linear operator *ℵ* that applies a mapping vector field *v* : *ℤ*^*d*^ → ℝ to a discrete image *I* : *ℤ*^*d*^ → *R* to perform an interpolation on the grid *G*_*v*_ = {*x* + *v*(*x*) | *x* ∈ *ℤ*^*d*^}, mathematically saying

(13) $$\begin{array}{c} \left(ℵI\right)\left(x\right)=\left(ℑ\right)\left(x+v\left(x\right)\right), \end{array}$$

with *ℑ* being linear interpolation, defined by

(14) $$\begin{array}{c} \left(ℑ\right)\left(I\right)\left(x\right)=\underset{\epsilon \in {\left\{0,1\right\}}^{d}}{\sum }{\text{c}}_{\epsilon }\left(x\right)I\left(\left\lfloor x_{1}\right\rfloor +\epsilon_{1},\left\lfloor x_{2}\right\rfloor +\epsilon_{2},\ldots ,\left\lfloor x_{d}\right\rfloor +\epsilon_{d}\right), \end{array}$$

with ⌊*z*⌋ being the integer part of real number *z* and {*z*} = *z* − ⌊*z*⌋ is the fractional part. The coefficient *c*_*ϵ*_(*x*) is defined as

(15) $$\begin{array}{c} c_{\epsilon }\left(x\right)=\overset{d}{\underset{i=1}{\prod }}\left(\epsilon_{i}+\left(1-2\epsilon_{i}\right)x_{i}\right). \end{array}$$

**Figure 5:**
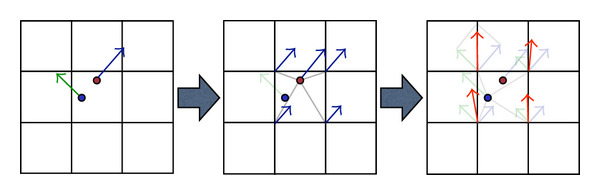
Geometrical conversion based on a splatting function with zero velocity field *v* (13). The method served as a bridge to transform the computation from an irregular grid to a regular grid which allows an efficient parallel implementation.

While the splatting operator was defined through a vector field, the splatting conversion from the irregular grid to the regular domain for an arbitrary input is defined as being a zero vector field.  Figure 5 displays the construction of a regular grid presentation of geometrical descriptors in 2D through splatting operator. The value at a grid point is computed by accumulating values interpolated at that point from its geometrical neighbors. Thus, closer neighbors will have more influence on the value of the point than farther points. In fact, we only need to consider the one-ring neighbors as farther points have a negligible contribution to its final value. We also assume that the field is continuous and smooth.

Though the splatting operator has a linear complexity in terms of the size of geometry descriptors, it is the performance bottleneck in practice. The single CPU thread-based splatting function is too slow for interactive applications. Even close discrete points do not share the same cache as the definition of a neighbor in 3D does not map to a neighbor in the linear CPU cache. The multithread-based CPU splatting, which assigns each thread a single geometrical element, however, has a resource-fighting problem. That is, when we integrate grid value from its neighbor submanifold elements, it is likely that there are several elements in the neighbor, and these elements, which are assigned different threads, may try to accumulate the grid value at the same time. GPU implementation also has to face with the resource-fighting problem.

We can apply mutex locking to resolve the conflict. However, it is inefficient with thousands of threads on GPUs. A better solution is based on atomic operations, which are guaranteed to complete without being interrupted by the actions of other threads. Currently, CUDA does not support atomic operations for floating point numbers but integer numbers. Here we propose two different approaches for splatting computation: the collision-free splatting scheme via a fast parallel sorting and the atomic splatting scheme using a fixed-point representation.

**Figure 6:**
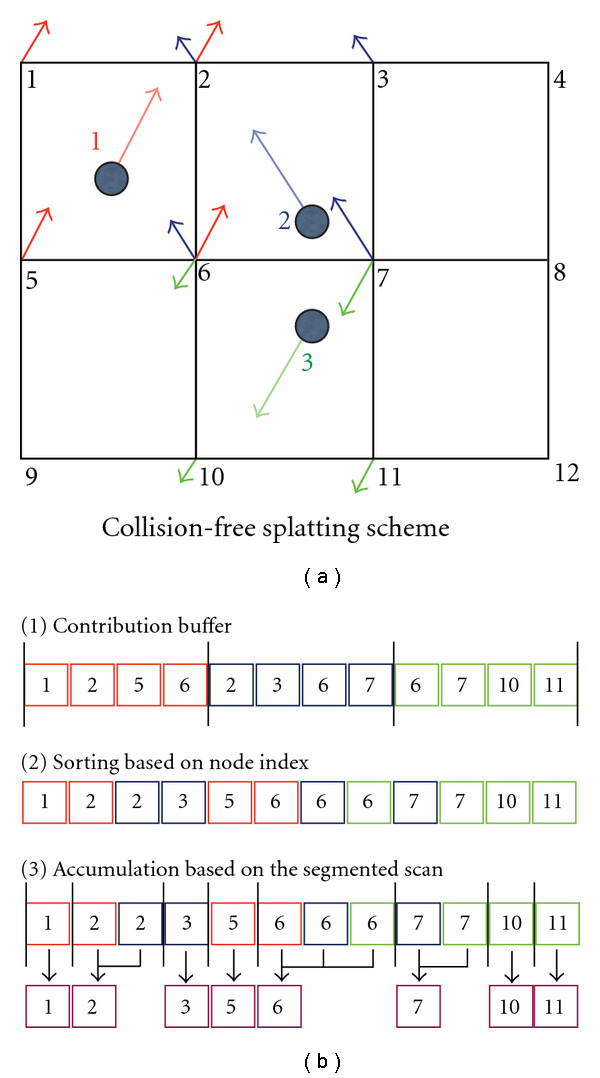
Collision-free splatting implementation using fast parallel sorting. The method is based on ordering the node contribution ID to resolve resource conflicts which allows a parallel efficient integration based on an optimal parallel prefix scan implementation.

The *collision-free splatting* scheme is applied for systems without any atomic operation support. As shown in Figure 6, we employ a fast parallel sorting to resolve the shared-resource fighting problem. The algorithm involves three steps.

- Compute the contribution of each geometrical descriptor to grid nodes.
- Sort the contribution based on node indexes. The contribution array is segmented based on node indexes.
- Apply a parallel segmented prefix sum scan [40] to integrate all node values.

All of these steps are implemented efficiently in parallel on the GPU. The first step is simply a pointwise computation. For the second step, we apply the fast parallel sorting [41]. The third step is performed using the optimal segmented scan function in the CUDA Performance Processing library (CUDPP) [40]. The sorting scheme on CUDA is a magnitude faster than an optimal multithreaded, multicore implementation on CPUs [29]. While this scheme is quite efficient and is the only solution on CUDA 1.0 devices, its performance largely depends on implementations of two essential functions: the parallel sorting and the segmented scan. Also the memory requirement of the method is proportional to the number of shooting points (which can be as large as the grid size) and the size of the neighbor (which is eight for 3D implementation). The memory usage become even worse as fast parallel sorting based on radix sorting that could not perform in-place but out-of-place sorting so the method requires another copy of the contribution array. In many circumstances, we found a better solution both in terms of performance and memory usage based on atomic operations supported on the CUDA 1.1 and later devices.

The *atomic splatting* scheme resolves the shared-resource fighting problem using atomic operations. While atomic floating point operations are currently not supported, it is possible to simulate this operation based on a fixed-point presentation. In particular, instead of accumulating the floating point buffer, we explicitly convert floating point values to integer representations through a scale. This allows the accumulation to be performed on integer buffers.

The parallel splatting accumulation is implemented by assigning each geometrical descriptor a GPU thread, which computes the contribution to the neighbor grid points based on its current value and distances to the neighbor grids. These floating point contribution values are then converted to integer presentation through a scale number, which is normally chosen as a power of two (we use 2^20^, in practice) so that a fast shifting function is sufficient to perform the scale. The atomic integer adding operator allows values to be accumulated atomically at each grid point concurrently from thousand of threads. In our implementation, the contribution computations—upscale and the integer accumulation steps—are merged to one processing kernel to eliminate (1) an extra contribution buffer, (2) extra memory bandwidth usage to store, reload, and rescale the contribution buffer from the global memory, and (3) the call overheads of the three different GPU processing kernel. The accumulation result is then converted back to floating value by the division to the same scale value.

We further amortize the performance on later generation of GPU devices using the atomic shared-memory operations, which are a magnitude faster than operations on GPU global memory. We exploit the fact that in diffeomorphic registration the velocity field is often smooth and show large coherence between neighbors, so it is likely that two close points will share the same neighbors. Thus, it would be better to accumulate the values of the shared neighbors in the shared memory instead of the global memory. We assign each block of threads a close set of splatting points and maintain a shared memory accumulation buffer between threads of the same block. The accumulation results on the shared memory are then atomically added to the accumulation buffer on the global memory. This approach exploits the fast atomic functions on the shared memory and at the same time reduces the number of global atomic operations. This optimization is especially effective on a dense velocity field, which shows significant coherency between neighbor points. 

#### 4.2.3. Interpolation

Even though the probabilistic and geometric descriptors are represented by independent data structures on separate domains, they are, in fact, different representatives of the same anatomical subject that is updated during ODE integration under the influence of the time-dependent velocity field along a registration evolution path. While the computation occurs on the regular grid, interpolation is necessary to maintain the consistency of multicompartment anatomies as they undergo deformation. Given a deformation *h*, we update probabilistic images using backward mapping and geometries using forward mapping (3).

**Figure 7:**
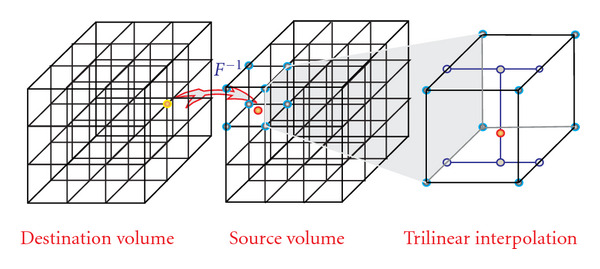
Reverse mapping based on 3D trilinear interpolation that eliminates the missing data of a forward mapping. The implementation on GPU exploits the hardware interpolation engine to achieve significant speedup.

**Figure 8:**
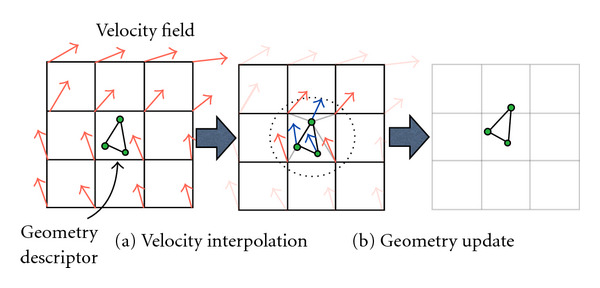
Geometries are updated through the interpolation from the velocity field. This step maintains the consistency between probabilistic and geometrical compartments of the mixture model.

 A computationally efficient version of ODE integration is the recursive equation that computes the deformation at time *t* based on the deformation at the time *t* − 1. That is, *h*_*t*_ = *h*_*t*−1_(*x* + *v*(*t* − 1)). This computation is done by a reverse mapping operator (Figure 7), which assigns each destination grid point a value interpolated from the source volume grid's neighbor points. The reason for using a reverse mapping operator instead of a forward mapping one is to avoid missing data values at the grid points that makes computation of forward mappings intractable. A reverse mapping requires the maintenance of reverse velocity fields. The update of geometric descriptors is based on a forward vector field derived by inverting direction of the reverse velocity field. Algorithmically, the probabilistic and geometric descriptors are updated in opposite directions. The updating process of geometric descriptors is illustrated in Figure 8.

While the selection of interpolation strategies such as 3D linear interpolation, cubic interpolation, high-order interpolation depends on the quality requirement of the registration, the updating process of both probabilistic and geometric descriptor needs to share the same interpolation strategy so that they are consistent with one another. In practice, 3D linear interpolation is the most popular technique because it is computationally simple and efficient and it can produce satisfactory results especially with large kernel width for currents norm. On GPUs, this interpolation process is fully hardware accelerated with 3D texture volume support from CUDA 2.0 APIs. Another optimization is based on the texture cache that helps improve the lookup time from the source volume due to large coherency in the diffeomorphic deformation fields. 

### 4.3. Other Performance Optimizations

Besides an optimized, parallel implementation for particle mesh computation, we further improve the performance with parallel surface normal and multiscale computation on GPUs. These optimizations keep the entire processing flow on GPUs, eliminating the need to transfer the data back and forth between CPU memory and GPU memory which is the main bottleneck for many GPU applications. 

#### 4.3.1. Parallel Surface Normal Computation on GPUs

While the geometrical descriptor involved in our registration framework was defined as a surface element (a triangle) with all property values on its vertices, the computation was defined at the centroid following its normal direction and weighted by the size of the surface element (11). This computation requires the computation of a weighted normal at the centroid of each surface element from the geometric descriptors. We perform this operation in parallel on the GPU by assigning each surface element a thread. We then employ the texture cache to load the geometrical data from global memory; while the neighbor triangle shared the same vertices, the loading values are highly likely in the cache and cost almost the same amount of time to access from the shared memory. We also store the three components of the normal in three separated arrays to allow coalesced access that gives better memory bandwidth efficiency. 

#### 4.3.2. Multiscale Computation on GPUs

**Figure 9:**
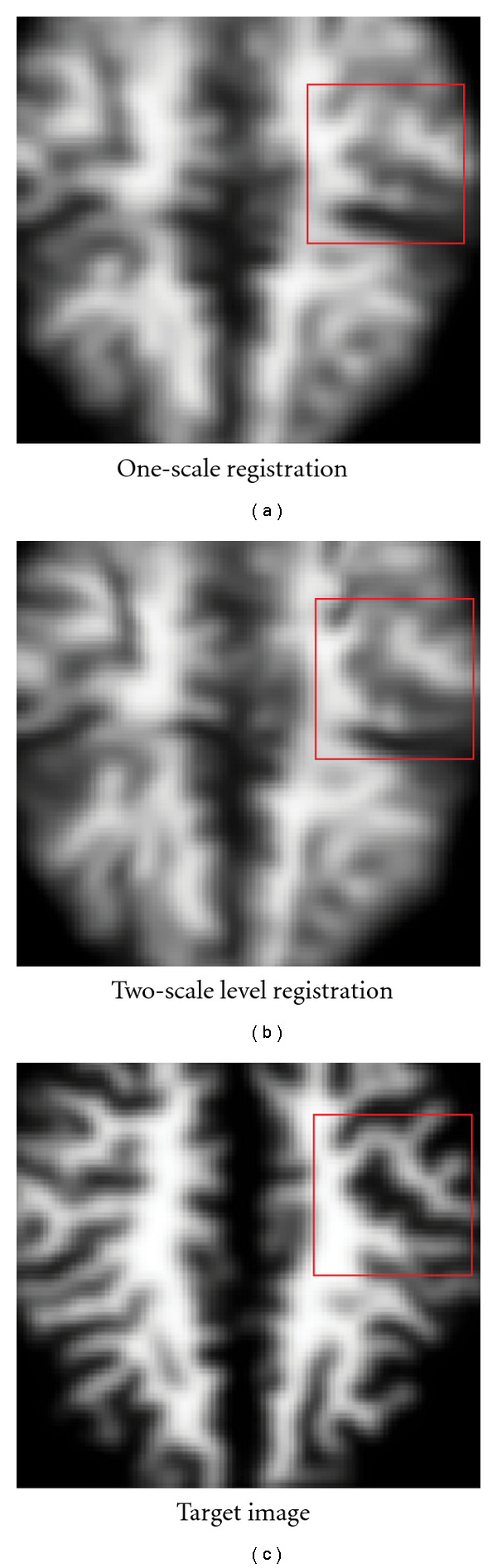
Multiscale registration using different sizes of computation kernels helps capture large- and small-scale changes in different levels and also increases the convergence rate of the algorithm.

Multiscale registration is an advanced registration technique to improve quality of the results by registering anatomies at different scale levels. The method also handles the local optimal matching of gradient-descent optimization. In our registration framework, the primary purpose of doing multiscale computation is to capture both the large changes in the shape and also the small changes as the registration anatomy converged to the target. The method effectively handles the nonlinear, localized shape changes, as is shown in Figure 9. It also serves as an effective method to increase the convergence rate and reduces the running time significantly. The challenge of applying multiscale computation is that there is no mathematical foundation for exact multiscale computation on a regular grid. The level-of-detail techniques (LOD) are the only approximations that gives no guarantee on the quality. Here, we achieve the multiresolution effect through changing the size of a registration kernel, such that we use a larger kernel width and step size to mimic the effect of large-scale and smaller kernel width and step size to capture the details. Our method did not require resampling of the grids, so there are no additional quantization errors. 

## 5. Results

For evaluation, we used an AMD Phenom II X4 955 CPU commodity system, 6 GB DDR3 1333, with NVIDIA GTX 260 GPU 896 MB. We quantify both aspects of the method: registration quality and performance. Runtime is measured in millisecond. 

### 5.1. Registration Quality

**Figure 10:**
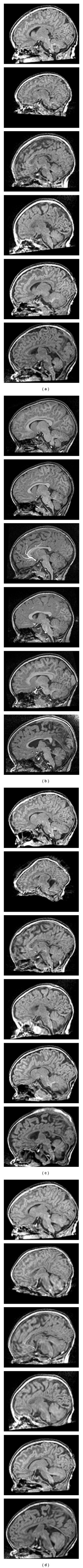
Registration results of neonates mapped to 2-year olds. From left to right: (a) neonatal T1 image after affine registration, (b) reference T1 image at 2 years, followed by (c) neonatal T1 after deformable mutual information registration using B-splines, and (d) after combined probabilistic and geometric registration. From top to bottom: subjects 0012, 0102, 0106, 0121, 0130, and 0146. We note that the initial affine registration for subject 0102 (second row, second column) is incorrect; however our method managed to compensate and generate improved result compared to deformable mutual information registration.

**Figure 11:**
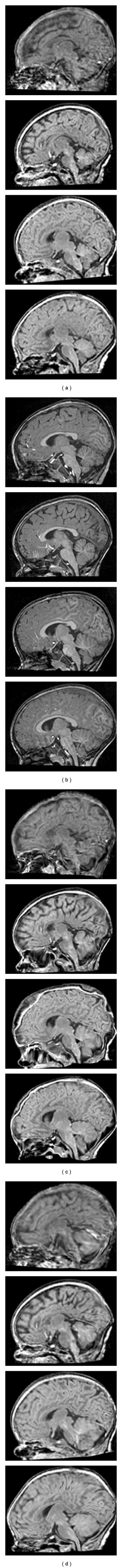
Registration results of neonates mapped to 2-year olds. From left to right: (a) neonatal T1 image after affine registration, (b) reference T1 image at 2 years, followed by (c) neonatal T1 after deformable mutual information registration using B-splines, and (d) after combined probabilistic and geometric registration. From top to bottom 0156, 0174, 0177, and 0180.

We have applied the registration method for mapping neonatal MRI scans to 2-year MRI scans of the same subjects in ten datasets. The datasets are taken from an ongoing longitudinal neuroimaging study with scans acquired at approximately two weeks, one year, and two years of age. Due to rapid early brain development, each longitudinal MR scan shows significant changes in brain size and in tissue properties. For comparison, we also applied the standard intensity-based deformable registration using mutual information (MI) metric and B-spline transformation proposed by Rueckert et al. [7], which has been applied for registering 1-year old and 2-year old infants [46]. Both deformable registration methods are initialized using the same global affine transformation generated using the mutual information metric. The T1-weighted images before and after registration using the different approaches for the first three subjects are shown in Figures 10 and 11.

 A quantitative study of the performance of the registration method is performed by measuring the overlap between the transformed segmentation maps of neonates to the segmentation maps of 2-year olds. Since we consider the segmentation maps at two years of age to be the standard, we use the following overlap metric:

(16) $$\begin{array}{c} \text{Overlap}\left(h\cdot S0,S2\right)=\frac{\left|h\cdot S0\cap S2\right|}{\left|S2\right|}, \end{array}$$

where *h* · *S*0 is the transformed neonate segmentation map, *S*2 is the reference 2-year segmentation map, and |·| indicates the volume of a binary map. We note that this metric gives considerably lower values for deviation from *S*2 than the standard Dice coefficient.  Table 1 shows the quantitative analysis for the brain parenchyma (a combination of white matter and grey matter) and cerebellum segmentation maps without registration, using standard MI registration, and our method. We use brain parenchyma since white matter and grey matter on their own are hard to distinguish in early developing brains. Registration using MI fails for parenchyma because it does not account for the two white matter distributions in neonates. Registration using both probabilistic and geometric descriptors provides better results and is generally more stable for the structures of interest. In particular, our method better preserves the shape of the cerebellum, which has weak intensity boundaries in regions where it touches the cerebrum and thus cannot be registered properly using only image-based information. Another significant challenge is that the cerebellum growth is distinctly different from the growth of neighboring structures. Using cerebellum boundary represented by currents, our method captures the growth better than MI registration. 

### 5.2. Performance

We quantify the performance with two critical steps in particle mesh approach: the splatting and the interpolation. We measured the performance with typical volume sizes.

**Splatting**

The splatting performance varies largely depending on the regularity of the deformation fields due to memory collision problem. Here we measured with three types of deformation fields: a random deformation, which maps points randomly over the whole volume, a diffeomorphic deformation, the typical type of deformation from the registration of brain images that we use in our framework, and a singular deformation, which collapses to a point in the volume.  Table 2 shows the runtime comparison in milliseconds of different splatting implementations mentioned in Section 4.2.2: CPU reference, collision-free sorting approach, atomic fixed-point operation, and atomic operation with shared memory.

The result shows that the performance gain of GPU approaches varies depending on the regularity of the deformation field inputs. The singular deformation has the lowest performance gain because most of the value accumulated to a small point neighbor hence parallel accumulation is greatly limited. Though having better performance gain, the random deformation spreads out in the whole volume that leads to ineffective caching (both in GPUs and CPUs). Fortunately, our atomic optimization with shared memory achieved the best performance gain with diffeomorphic deformation which we used in practice. The main reason is that the diffeomorphic deformation shows large coherence between neighbor points that allows more effective caching through GPU shared memory. The collision-free approach based on sorting shows stable performance since it is independent from the memory collision of other approaches.

**Interpolation**

Table 3 shows the runtime comparison in milliseconds of different 3D interpolation implementations: CPU reference, simple approach (GPU global memory), linear 1D texture, and 3D texture.

The interpolation runtime shows that reverse mapping using the accelerated hardware achieves the best performance and is about 38x faster than CPU reference implementation on the evaluation hardware. However, this method suffers from lower floating point accuracy. To not further introduce more errors to the approximation, we apply the 1D-linear texture-cache implementation instead which is as fast as the accelerated hardware but retains the floating point precision. The method produces results equivalent to the CPU reference.

**Overall Performance**

We have also compared the performance between our method and the standard MI registration. Registration using our approach on the GPU takes 8 minutes on average, while registration on the CPU using mutual information metric and B-spline transformation takes 100 minutes on average. Detailed time measures are listed in Table 4.

Overall, computing the currents norm and its gradient between a surface with 160535 triangular faces and another with 127043 faces takes approximately 504 seconds on CPU, while it takes 0.33 seconds with our GPU implementation. The speed gain is in order of three magnitudes over the equivalent CPU implementation using particle mesh, while the computing time for the exact norm on CPU is difficult to measure since it takes significantly longer. The proposed algorithm typically converges in 1000 iterations, so on average it takes less than eight minutes to register two anatomies. This allows us to perform parameter exploration and real-time analysis on a single desktop with commodity GPU hardware. 

## 6. Conclusions

We have proposed a registration framework that makes use of the probabilistic and geometric structures of anatomies embedded in the images. This allows us to enforce matching of important anatomical features represented as regional class posteriors and tissue boundaries. Our framework allows us to register images with different contrast properties by using equivalent anatomical representations, and we have demonstrated results for registering brain MRIs with different white matter appearances at early stages of growth. The overlap validation measures in Table 1 show that geometric constraints, particularly for the cerebellum, are crucial for registering structures undergoing significant growth changes.

In the future, we plan to apply this framework in early neurodevelopmental studies for analyzing the effects of neurological disorders such as autism and fragile X syndrome. The proposed registration framework is generic and independent of the application domain; it can thus be applied to any registration where one encounters large-scale deformation and different appearance patterns. We also want to incorporate other submanifolds representations and their computation such as point sets (*ℳ*(0)) and curves (*ℳ*(1)). Such additional representations are potentially critical in clinical applications involving anatomical landmark points (e.g., anterior commissure and posterior commissure) as well as curve structures (e.g., blood vessels, sulcal lines, white matter fiber tracts). All these computations can be done efficiently and entirely on GPUs and potentially will improve the results by guiding the registration process to preserve critical geometries. The efficiency of the GPU method also provides an opportunity to apply the algorithm for high-quality atlas formation using our framework on a GPU cluster, which gives us the ability to perform statistical tests that are previously impossible due to excessive time requirements. 

## Figures and Tables

**Table 1:** Overlap measures comparing the registered segmentation maps against the reference segmentation maps for the parenchyma and cerebellum structure, obtained through without deformation (None), deformable mutual information registration (MI), and our proposed method (P + G).

	Subject	0012	0102	0106	0121	0130	0146	0156	0174	0177	0180
Parenchyma	None	0.829	0.545	0.813	0.833	0.921	0.750	0.818	0.837	0.782	0.707
	MI	0.799	0.449	0.754	0.777	0.902	0.708	0.780	0.832	0.774	0.687
	P + G	0.903	0.883	0.884	0.868	0.881	0.860	0.875	0.879	0.913	0.874

Cerebellum	None	0.573	0.263	0.506	0.506	0.638	0.555	0.535	0.503	0.526	0.593
	MI	0.755	0.212	0.588	0.515	0.732	0.820	0.713	0.569	0.631	0.777
	P + G	0.881	0.821	0.875	0.878	0.858	0.899	0.907	0.885	0.896	0.892

**Table 2:** Runtime comparison, in milliseconds, of different splatting implementations on volume sized 144 × 192 × 160 and 160 × 224 × 160 using collision-free sorting approach, atomic operation with fixed point presentation, atomic operation on the shared memory and CPU reference.

Size	Method	CPU	Sorting	Atomic	Atomic shared
144 × 192 × 160	Random	826	105	29	30
	Diffeomorphic	331	110	105	14
	Singular	224	105	40	41

160 × 224 × 160	Random	1435	215	75	76
	Diffeomorphic	775	224	152	21
	Singular	347	215	144	144

**Table 3:** Runtime comparison, in milliseconds, of different 3D interpolation implementations for reverse mapping operator without memory caching (GPU global), with linear texture cache (1D linear) and hardware accelerated interpolation using 3D texture. The GPU-accelerated implementation is about 40 times faster than CPU reference and gives identical results.

Method	CPU	GPU global	1D linear	3D texture
256 × 256 × 256	777	30	24	19
160 × 224 × 160	209	10.4	7.3	6.8
144 × 192 × 160	173	6.8	4.8	5.4
160 × 160 × 160	149	6.6	5.0	5.2

**Table 4:** Time elapsed, in minutes, for registration using deformable mutual information (MI) on the CPU (AMD Phenom II X4 955, 6 GB DDR3 1333) and our proposed approach (P + G) on the GPU (NVIDIA GTX 260, 896 MB) with 1000 iterations of gradient descent.

Subject	0012	0102	0106	0121	0130	0146	0156	0174	0177	0180
MI on CPU	92	63	103	92	101	112	106	99	91	96
P + G on GPU	9	8	8	8	8	7	9	8	7	7
